# Supplementary figures and images for: Diversity and spatiotemporal variations in bacterial and archaeal communities within Kuwaiti territorial waters of the Northwest Arabian Gulf
Source: PLoS One. 2023 Nov 16;18(11):e0291167. doi: 10.1371/journal.pone.0291167 (PMC10653540; doi:10.1371/journal.pone.0291167)

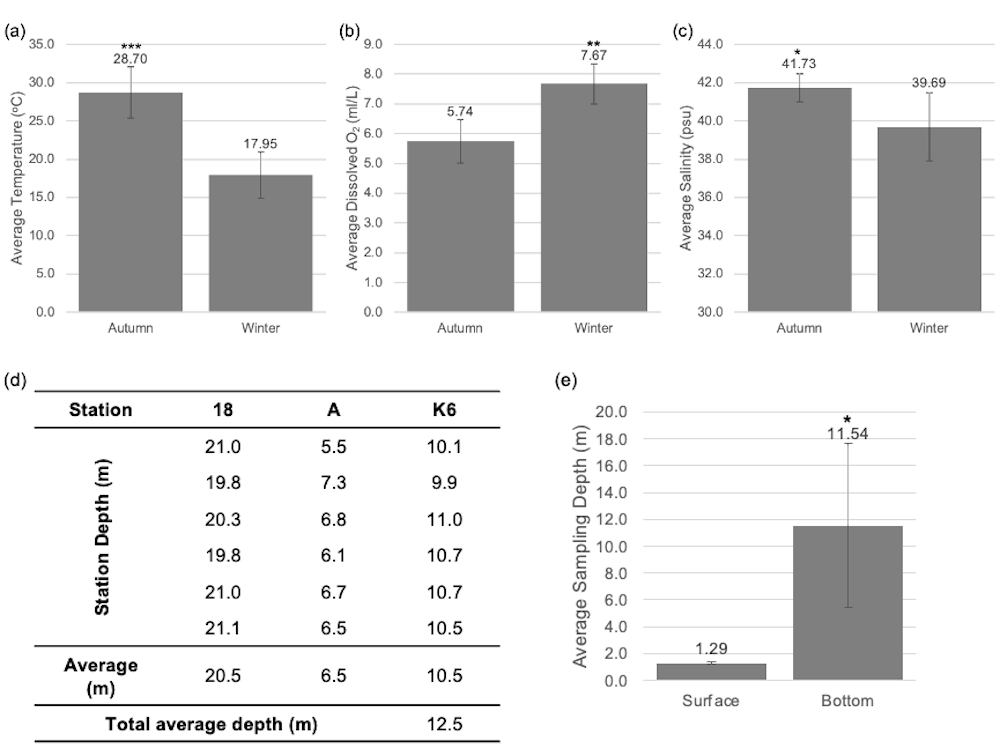

Supplement: S1 Fig — (a-c) Variations in average levels of measured physicochemical parameters between autumn and winter. Average values of seawater (a) temperature, (b) dissolved oxygen levels, and (c) salinity levels were compared between the autumn (n = 18) and winter (n = 18) seasons. Error bars represent standard deviations. *p<1E-4, **p<1–8, ***p<1E-11 (Student’s t-test). (de) Station depth and sample acquisition depth. (d) Total station depth measured during each of the six months of sampling for each of the three stations (K6, 18, and A). (e) Average depth of sample acquisition for surface (n = 18) and bottom (n = 18) samples. Error bars represent standard deviations. *p<1–7 (Student’s t-test). (PNG) [file pone.0291167.s001.png]

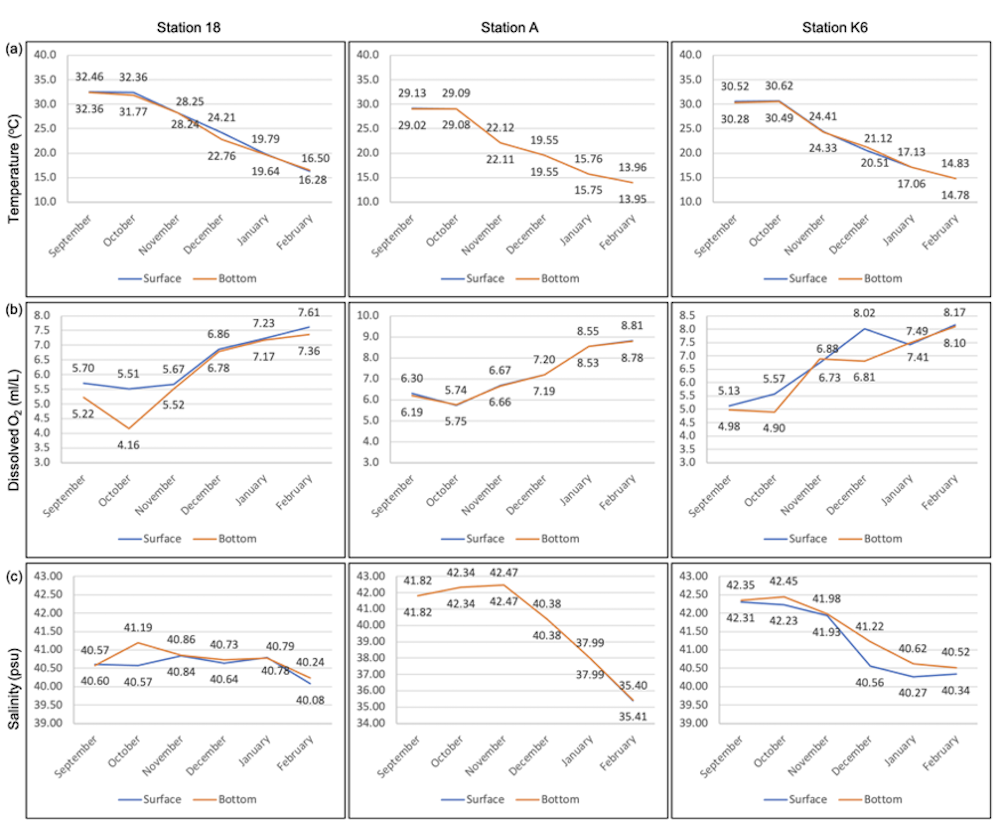

Supplement: S2 Fig — Values of seawater (a) temperature, (b) dissolved oxygen levels, and (c) salinity levels were measured at the time of sampling at both the surface (blue line) and bottom (red line) of each of the three sampling stations (Station 18 (left), Station A (middle), and Station K6 (right)). Values for each measured parameter are depicted for each month of the six-month sampling period (September 2019–February 2020). (PNG) [file pone.0291167.s002.png]

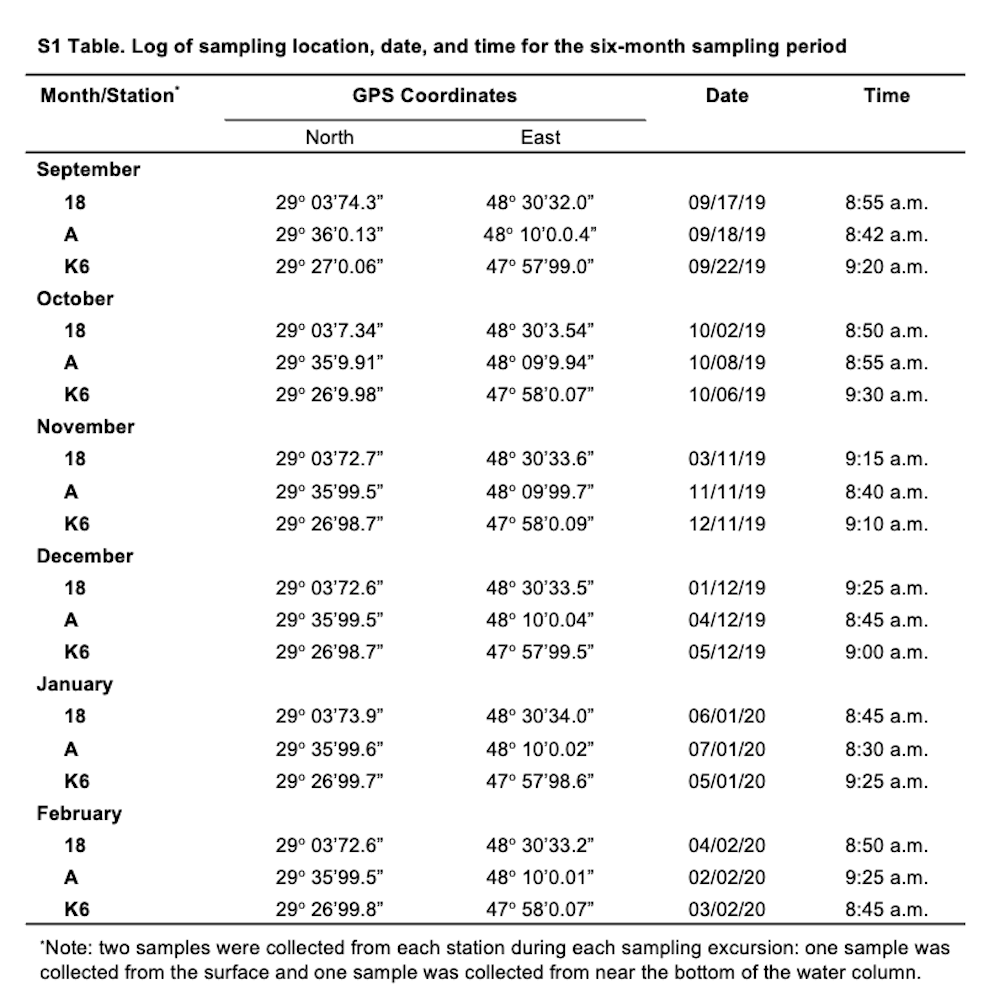

Supplement: S1 Table — (PNG) [file pone.0291167.s003.png]

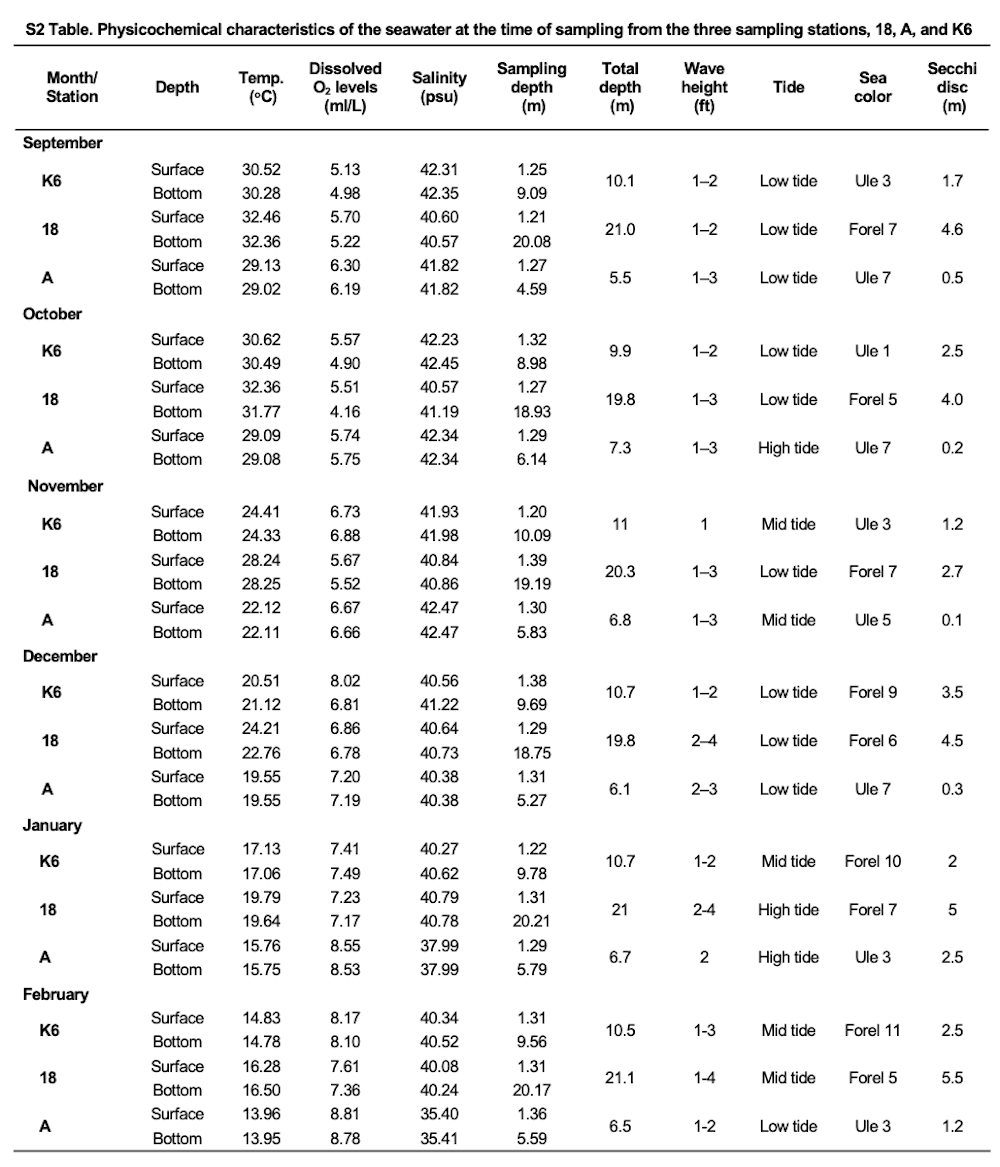

Supplement: S2 Table — (PNG) [file pone.0291167.s004.png]

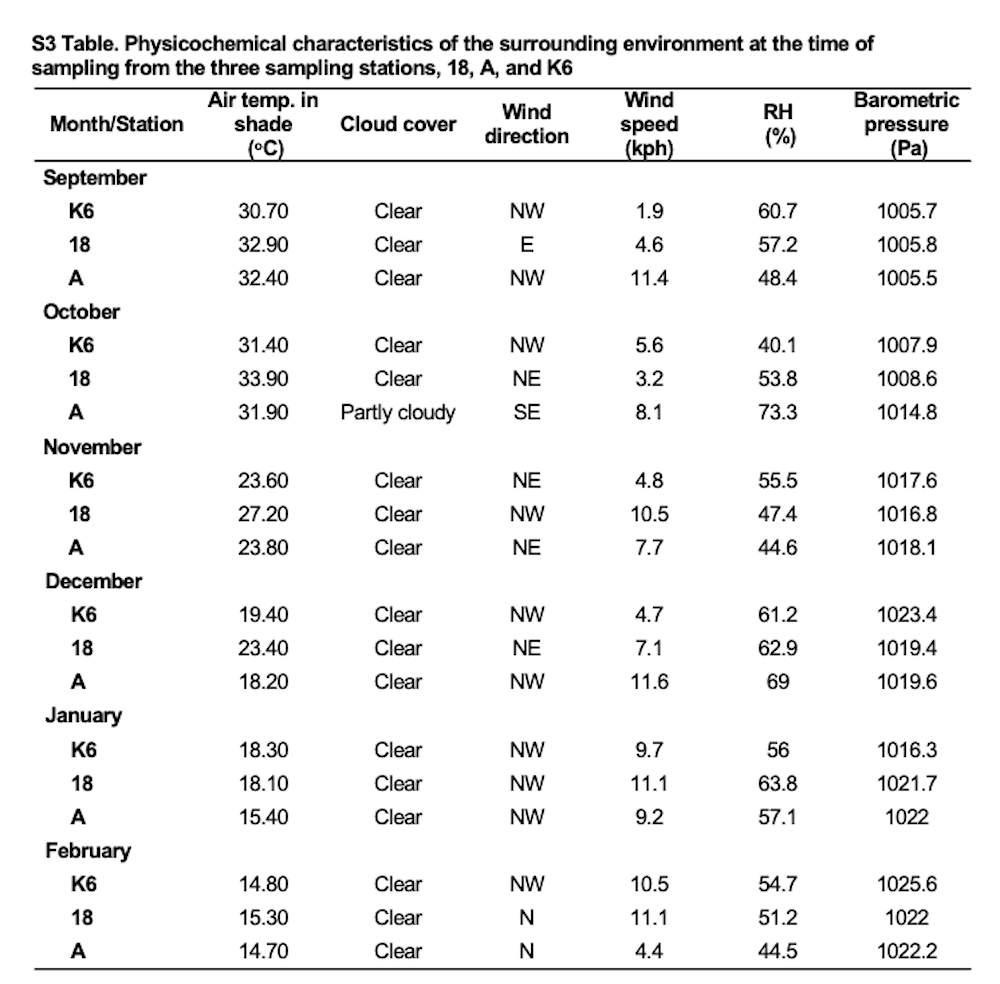

Supplement: S3 Table — (PNG) [file pone.0291167.s005.png]

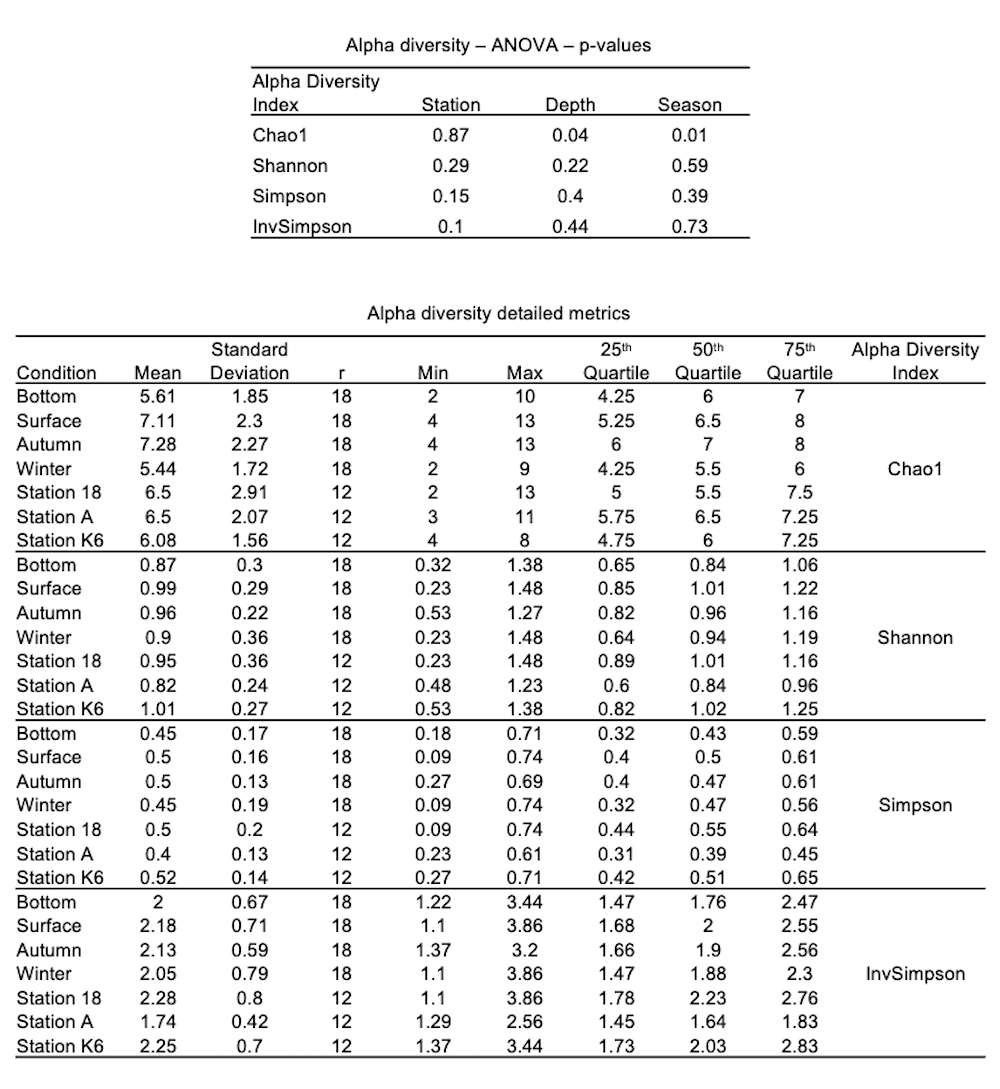

Supplement: S4 Table — (PNG) [file pone.0291167.s006.png]
