# Supplementary material for: Diversity and spatiotemporal variations in bacterial and archaeal communities within Kuwaiti territorial waters of the Northwest Arabian Gulf
Source: PLoS One. 2023 Nov 16;18(11):e0291167. doi: 10.1371/journal.pone.0291167 (PMC10653540; doi:10.1371/journal.pone.0291167)
Supplement: Attachment — Submitted filename: Response to Reviewers_073023.docx [file pone.0291167.s039.docx]

Dear Dr. Santos,

Thank you kindly for your message. A response to the latest query sent on July 24, 2023, is provided below in bold, blue font.

1. *Please note that PLOS ONE is unable to publish previously copyrighted maps or satellite images, or images created using proprietary data. For these reasons, we cannot publish images generated by software which copyrights their output (such as Google Maps, Street View, and Earth). In order to use these images in your submission, we require explicit permission from the copyright owner to publish the figures under the CC BY 4.0 license.

   At this time, please kindly clarify the following regarding Figure 1:

   a) Where did the author of this image obtain the map, basemap, shapefiles, map data, etc. in Figure 1?*

**The author of the image used in the originally submitted Figure 1 is Dr. Takahiro Yamamoto, a colleague in the Oceanography Research Group at the Kuwait Institute for Scientific Research (email:** [tyamamoto.kisr@gmail.com](mailto:tyamamoto.kisr@gmail.com); [tyamamoto@kisr.edu.kw](mailto:tyamamoto@kisr.edu.kw)**). Dr. Yamamoto obtained the basemap for that image from DIVA-GIS, a free geographic information system software program (**<https://www.diva-gis.org/>**). He then used the ArcGIS software (**<https://www.arcgis.com/>**) to annotate the map and generate the version used in the original Figure 1. Dr. Yamamoto newly generated that figure in June 2023 upon my request and specifically for my use.**

**That being said, the aforementioned figure/map was included in a chapter that I submitted in June for publication in an upcoming book. Thus, to avoid any potential conflicts arising from use of the same figure in the book chapter and in this manuscript, an alternative figure has been uploaded for use in this manuscript.**

**The new figure was recently kindly generated by Dr. Yamamoto. For the new image (currently submitted as Figure 1), Dr. Yamamoto obtained the basemap from Landsat (**<http://landsat.visibleearth.nasa.gov/>**), one of the resources that was recommended by PLOS ONE. The images used were obtained on 2023-04-03 and 2023-04-04. Once again, Dr. Yamamoto used the ArcGIS software (**<https://www.arcgis.com/>**) to annotate the map and generate the final version used in the updated Figure 1.**

**The caption for the replacement figure has been updated with source information and currently reads: “Fig 1. Sampling site description. Map showing northwest region of the Arabian Gulf bordering Kuwait City, specifically the water body known as Kuwait Bay (basemap obtained from Landsat (**[**http://landsat.visibleearth.nasa.gov/**](http://landsat.visibleearth.nasa.gov/)**) and annotated using the ArcGIS software (**[**https://www.arcgis.com/**](https://www.arcgis.com/)**). Red dots indicate the three sampling stations utilized for this study (Station A to the northeast of Kuwait Bay, Station K6 located inside Kuwait Bay, and Station 18 located to the southeast of Kuwait Bay). Figure courtesy of T. Yamamoto, Kuwait Institute for Scientific Research”.**

*b) Please state whether the map, basemap, shapefiles, map data, etc. have been previously copyrighted to your knowledge.*

**To the best of our knowledge, there are no copyrights associated with the newly generated figure/map.**
 *c) If any of the map, basemap, shapefiles, map data, etc. in this image have been previously copyrighted, we require specific consent from the copyright holder to publish these images in PLOS ONE, under the CC BY 4.0 license.*

**N/A**
